# Prognostic value of poly-microorganisms detected by droplet digital PCR and pathogen load kinetics in sepsis patients: a multi-center prospective cohort study

**DOI:** 10.1128/spectrum.02558-23

**Published:** 2024-03-25

**Authors:** Yuanhan Zhao, Ke Lin, Haocheng Zhang, Yanliang Zhang, Shaling Li, Shengguo Zhang, Wei Zhang, Aiming Zhou, Yangyang Zhuang, Jie Chen, Caixia Wu, Wei Zhou, Xiaoju He, Qiaoyan Yue, Meng Zhang, Yan Huang, Liang Li, Liang Hong, Fujing Cai, Lisu Huang, Zhengshang Ruan, Shanshan Xu, Yan Zhang, Xiaohua Chen, Jie Chen, Ying Ye, Tingting Bian, Jiabin Li, Jun Yin, Xiang Li, Lijing Jiang, Chen Lei, Jun Liu, Ying Zhang, Jialin Jin, Jingwen Ai, Jingye Pan, Wenhong Zhang

**Affiliations:** 1 Department of Infectious Diseases, National Medical Center for Infectious Diseases, Shanghai Key Laboratory of Infectious Diseases and Biosafety Emergency Response, Huashan Hospital, Fudan University, Shanghai, China; 2 Cancer Hospital of the University of Chinese Academy of Sciences (Zhejiang Cancer Hospital), Institute of Cancer and Basic Medicine (IBMC), Chinese Academy of Sciences, Hangzhou, China; 3 Department of Infectious Diseases, The Nanjing Hospital of Chinese Medicine affiliated to Nanjing University of Chinese Medicine, Nanjing, Jiangsu, China; 4 Department of Infectious Diseases, Xiangya Hospital Central South University, Changsha, Hunan, China; 5 Department of Infectious Diseases, The Third Affiliated Hospital of Wenzhou Medical University, WenZhou, Zhejiang, China; 6 Society of Clinical Epidemiology and Evidence-Based Medicine, Shanghai Medical Association, Shanghai, China; 7 Department of Intensive Care Unit, The First Affiliated Hospital of Wenzhou Medical University, Wenzhou, Zhejiang, China; 8 Department of Infectious Disease, Xinhua Children’s Hospital, Xinhua Hospital, Shanghai Jiao Tong University School of Medicine, Shanghai, China; 9 Department of Anesthesiology and Surgical Intensive Care Unit, Xinhua Children’s Hospital, Xinhua Hospital, Shanghai Jiao Tong University School of Medicine, Shanghai, China; 10 Department of Infectious Diseases, Shanghai Sixth Hospital, Shanghai Jiaotong University, Shanghai, China; 11 Department of Infectious Diseases, The First Affiliated Hospital of Anhui Medical University, Hefei, Anhui, China; 12 Department of Critical Care Medicine, Minhang Hospital, Fudan University, China, Shanghai; 13 Department of Laboratory, Wuxi No.5 People’s Hospital Affiliated to Nantong University, Nantong, China; 14 Shanghai Huashen Institute of Microbes and Infections, Shanghai, China; Beijing Institute of Genomics, Chinese Academy of Sciences, Beijing, China

**Keywords:** sepsis, droplet digital polymerase chain reaction, pathogen load, polymicrobial infection, mortality

## Abstract

This study aimed to investigate the prognostic value of a novel droplet digital polymerase chain reaction (DDPCR) assay in sepsis patients. In this prospective cohort study, univariable and multivariable Cox regressions were used to assess risk factors for 28-day mortality. We also monitored pathogen load together with clinical indicators in a subgroup of the cohort. A total of 107 sepsis patients with positive baseline DDPCR results were included. Detection of poly-microorganisms [adjusted hazard ratio (HR) = 3.19; 95% confidence interval (CI) = 1.34–7.62; *P* = 0.009], high Charlson Comorbidity Index (CCI) score (adjusted HR = 1.14; 95% CI = 1.01–1.29; *P* = 0.041), and Sequential Organ Failure Assessment (SOFA) score (adjusted HR = 1.18; 95% CI = 1.05–1.32; *P* = 0.005) at baseline were independent risk factors for 28-day mortality while initial pathogen load was not associated (adjusted HR = 1.17; 95% CI = 0.82–1.66; *P* = 0.385). Among 63 patients with serial DDPCR results, an increase in pathogen load at days 6–8 compared to baseline was a risk factor for 28-day mortality (*P* = 0.008). Also, pathogen load kinetics were significantly different between day-28 survivors and nonsurvivors (*P* = 0.022), with a decline overtime only in survivors and an increase from days 3 and 4 to days 6–8 in nonsurvivors. Using DDPCR technique, we found that poly-microorganisms detected and increased pathogen load a week after sepsis diagnosis were associated with poor prognosis.

**IMPORTANCE:**

This prospective study was initiated to explore the prognostic implications of a novel multiplex PCR assay in sepsis. Notably, our study was the largest cohort of sepsis with droplet digital polymerase chain reaction pathogen monitoring to date, allowing for a comprehensive evaluation of the prognostic significance of both pathogen species and load. We found that detection of poly-microorganisms was an independent risk factors for 28-day mortality. Also, pathogen load increase 1 week after sepsis diagnosis was a risk factor for 28-day mortality, and differential pathogen load kinetics were identified between day-28 survivors and nonsurvivors. Overall, this study demonstrated that pathogen species and load were highly correlated with sepsis prognosis. Patients exhibiting conditions mentioned above face a more adverse prognosis, suggesting the potential need for an escalation of antimicrobial therapy.

Registered at ClinicalTrials.gov (NCT05190861).

## INTRODUCTION

Sepsis is a life-threatening disorder that causes organ failure, which results from an unbalanced host response to an infection (1). There is significantly high risk of mortality in sepsis, considered one of the top causes worldwide (2). As reported, bacterial burden and the duration of infection could influence the outcome of patients with sepsis. Therefore, early identification of the pathogens is crucial for timely treatment of directed antibiotics application, and proper subscription of antibiotics would improve clinical outcomes (3, 4). However, currently, identifying and monitoring sepsis process still relies heavily on the patient's clinical representations, which, to some extent, might not be timely nor specific enough. Besides, traditional blood culture (BC), of which the results usually call for 2–5 days, could only provide results without quantitative results (5–7). More importantly, dynamically monitoring pathogen load is necessary for disease surveillance, considering the rapid changes in sepsis patients' condition, and the most direct and evident way to measure the efficacy of antimicrobial therapy would be evaluating the pathogen load, ideally combined with therapeutic drug monitoring.

Recently, droplet digital polymerase chain reaction (DDPCR), a novel technique of high sensitivity and capable of absolutely quantifying target molecules (8), has been expected to help with the diagnosis of bloodstream infections (BSIs) by rapidly detecting pathogens and load quantification. Earlier studies have reported the application value of DDPCR in BSIs and sepsis, as the assay represented high accuracy and sensitivity compared to traditional BC (9–12). Furthermore, several studies have recently explored the relationship between sepsis prognosis and the type of pathogen as well as quantitative information detected by the DDPCR assay (13, 14) in small sample sizes. Further researches in more general and larger population are needed. Therefore, we set out a multi-center prospective study to investigate whether the pathogen species and pathogen load detected by DDPCR were associated with mortality and to explore the indicators between survivors and nonsurvivors in sepsis.

## MATERIALS AND METHODS

### Patients and study design

**Fig 1:**
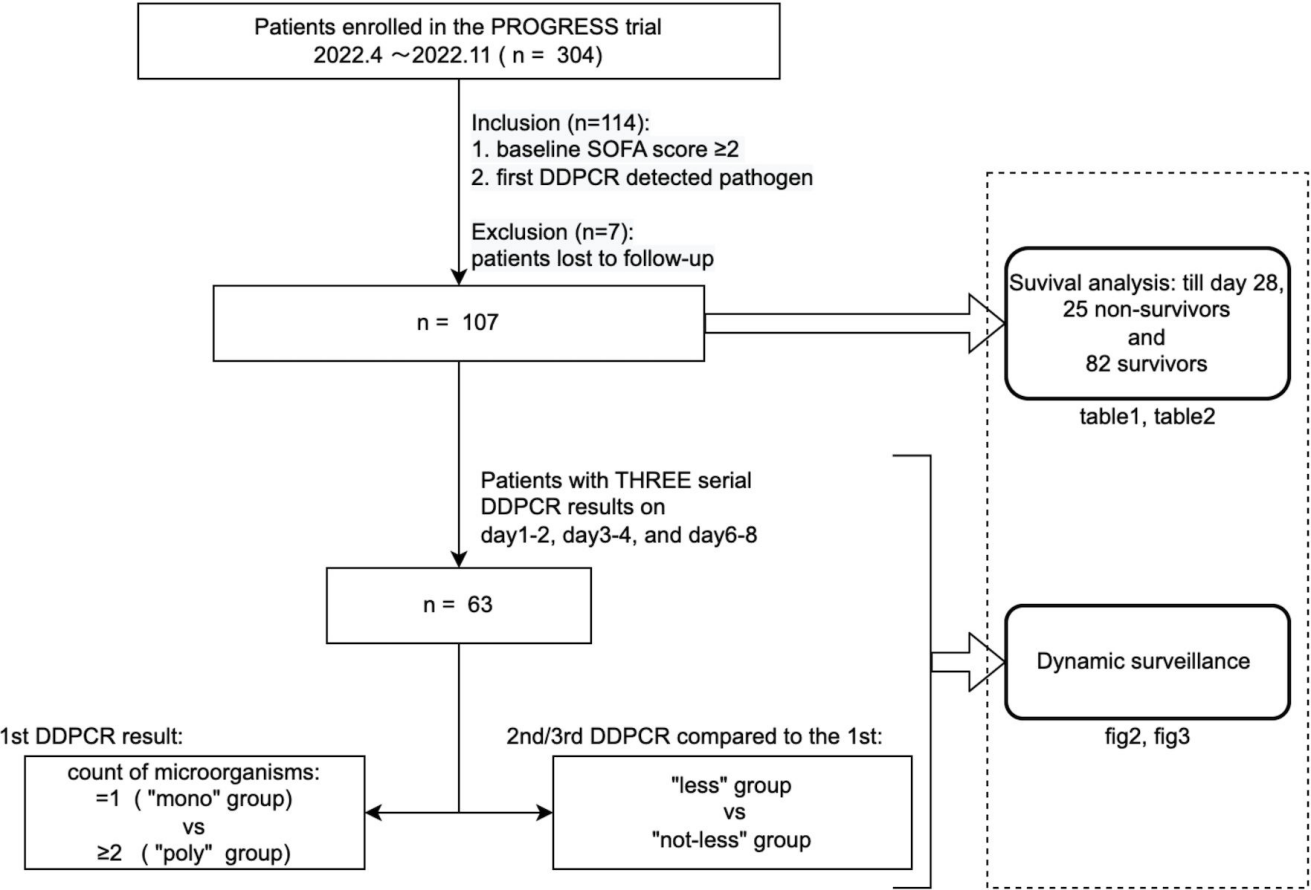
Flow chart of the study.

We studied patients who were enrolled in the parent PROGRESS trial between April 2022 and November 2022 (Fig. 1). The parent PROGRESS cohort, as a multicenter trial, is set in the infectious diseases departments and intensive care units (ICUs), and the main inclusion criteria are (1) 18 years old or older, (2) meet at least two of the systemic inflammatory response syndrome criteria, (3) hospitalized patients who have BC drawn on the same day as enrollment, and (4) written informed consent. The trial was approved by the Huashan Hospital Ethical Committee (protocol ID 2021-KY790) and has been registered on ClinicalTrial.gov under grant number NCT05190861.

We conducted the analysis on patients who met the following criteria: (1) baseline Sequential Organ Failure Assessment (SOFA) score not less than 2, meeting the sepsis 3.0 diagnosis criteria, and (2) the first DDPCR test at enrollment detected pathogen in the blood. Seven patients were excluded for loss to follow-up before day 28 or observed death events, thus leaving 107 patients as the total cohort in this study (Fig. 1).

We further analyzed the changes of the pathogen load and other clinical parameters in a subgroup of 63 patients who had three serial DDPCR test results: the first at enrollment on days 1 and 2 and the second and the third at two follow-up time points, that is, days 3 and 4 and days 6–8 (Fig. 1).

### Data collection

The patients with positive DDPCR results at enrollment had blood collected for monitoring on days 3, 7, and 7*n* (*n* > 1, that is, every 7 days, until there was an outcome). Synchronized BCs were drawn at enrollment with the first DDPCR. Apart from microbial tests, follow-up examinations were conducted to calculate the SOFA score as a measure of disease severity, as well as inflammatory markers including serum C-reactive protein and procalcitonin (PCT). We collected clinical data of enrolled patients through a uniformly designed case report form at enrollment and each follow-up points.

### Definitions

**Fig 2:**
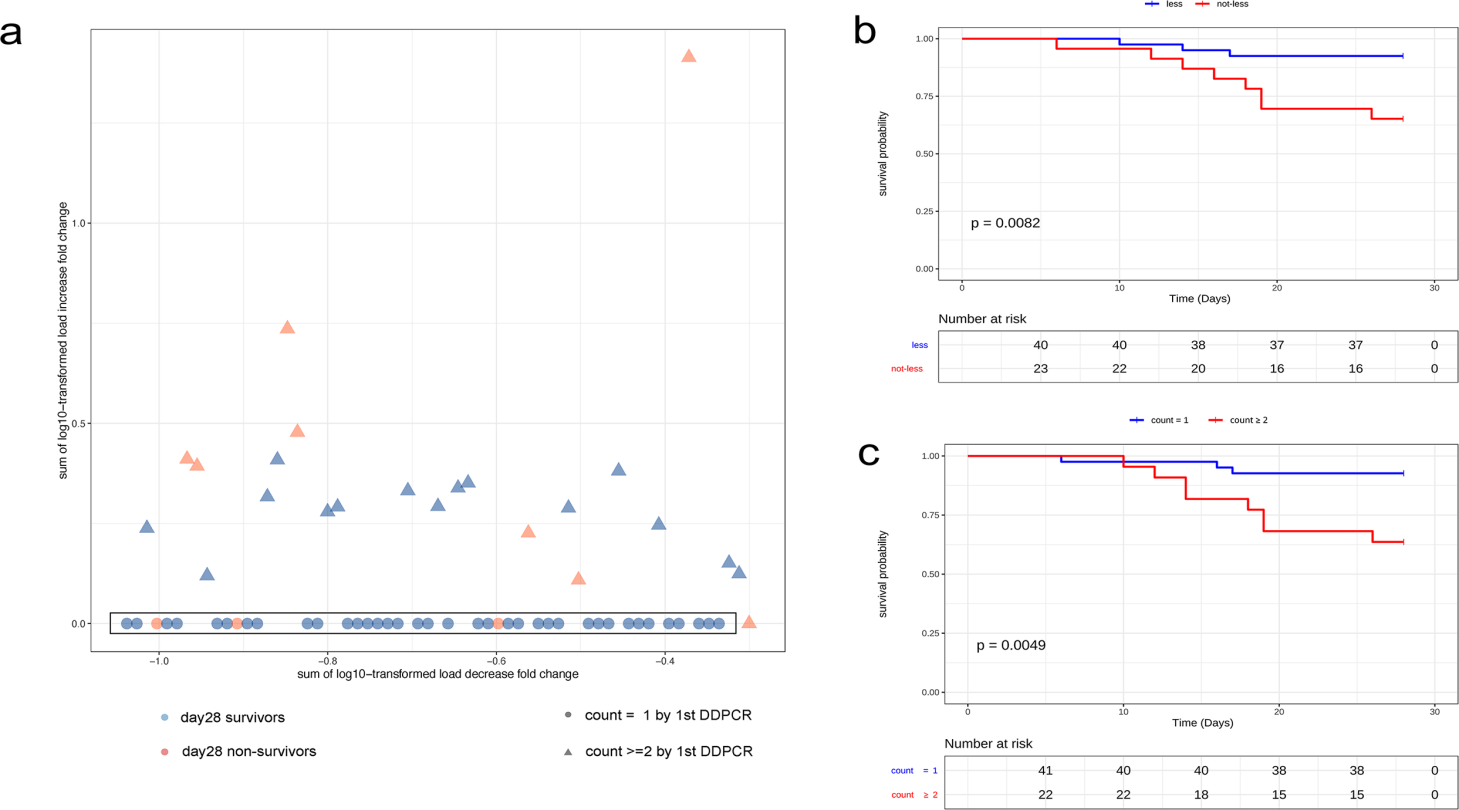
Relationship between pathogen load change monitored by the DDPCR assay and 28-day mortality. (**a**) Scatter plot showing the decrease and increase of pathogen DNA load detected by DDPCR of 63 patients. Each point represents a patient. Red points represent day-28 nonsurvivors (*n* = 11) and blue points represent survivors (*n* = 52). The circles represent the first DDPCR detection of mono-microorganism (count = 1; *n* = 41), and the triangles represent the detection of poly-microorganisms (count ≥2; *n* = 22). The coordinates of each point are (**X, Y**) and *x* represents the logarithmic sum of the load fold change decrease value (log10-transformed) of each patient. Therefore, the greater the reduction is, the farther the point moves to the left. The *y* axis represents the logarithmic sum of the load fold change increase value of each patient; therefore, the greater the increase is, the higher the position is. The points with *x* < 0 and *y* = 0 (in the black box) corresponding to the patients whose DDPCR test on days 6–8 only had a decrease in DNA load compared with the first DDPCR, and were defined as the “less group” (*n* = 40), while the points outside the black box corresponded to patients with no load change or increased load, which were defined as the “not-less group” (*n* = 23). (**b**) Kaplan–Meier curve showing overall survival in patients of the “less group” (*n* = 40) and the “not-less group” (*n* = 23). The *P* value was from log-rank test. (**c**) Kaplan–Meier curve showing overall survival in patients of the “mono group” (count = 1; *n* = 41) and the “poly group” (count ≥2; *n* = 22). The *P* value was from Breslow test and the log-rank *P* = 0.0041.

The DDPCR result was considered positive if one pathogen or more was detected. The patients who had one pathogen detected by the first DDPCR were defined as the “mono” group (*n* = 41), and the patients who had ≥2 species of pathogen detected were the “poly” group (*n* = 22), who were also mentioned as having polymicrobial infection. Also, by comparing the results of follow-up DDPCR at days 6–8 with the first one, patients can be divided into those with only decreased pathogen load for all pathogens detected by the first DDPCR (the “less” group; *n* = 40) and those with no load decrease or increase (the “not-less” group; *n* = 23) (Fig. 1 and 2a).

### The DDPCR assay

The digital PCR BSI assay can detect over 15 pathogens, which were selected from the most common bacteria and fungi detected in BSIs according to the China Antimicrobial Surveillance Network 2021 Report (CHINET) (15), and seven antimicrobial resistance genes. The tests were performed on a 6-fluorescent-channel DDPCR system [Pilot Gene Technology (Hangzhou) Co., Ltd., Hangzhou, China]. The targeting pathogens are listed in Table S2. First, the patient's peripheral venous blood samples were collected and centrifuged at 1,200 *g* for 5 minutes to separate the plasma, and cell-free DNA (cfDNA) extraction was performed using a magnetic bead method with 2 µL of plasma. Second, 10 µL of the DDPCR premix, which included detection primers, probes, and the necessary components for PCR amplification, was mixed gently with 5 µL of the cfDNA and loaded into a ready-to-use disposable chip. About 20,000 water-in-oil emulsion droplets were generated inside the chip by a droplet generator (DG32; Pilot Gene Technology) and were then amplified in a thermal cycler (TC1; Pilot Gene Technology). After PCR amplification, chips were loaded into a scanner (CS7; Pilot Gene Technology) for fluorescence signal reading. Finally, the software (GenePMS; Pilot Gene Technology) outputs the concentration value (copies/µL) for each target and the unit can be converted to copies per milliliter (copies/mL).

### Statistical analysis

The categorical data are expressed as frequencies and proportions (%), and continuous variables are expressed as medians and interquartile ranges (IQR) in the tables. All categorical variables were compared between the survivors and nonsurvivors using the Fisher's exact test or χ^2^ test, whichever is appropriate, and continuous variables were compared using the student *t*-test or the Mann–Whitney U test for *P* values presented. The pathogen cfDNA load, as a continuous variable, was calculated by summing the load of all detected microorganisms when poly-microorganisms were detected.

Univariable and multivariable analyses using Cox proportional hazards models were performed for the estimation of prognostic factors, with a time from admission of the PROGRESS trial to the death event or day-28 follow-up. The adjusted hazard ratio (HR) and 95% confidence intervals (CIs) were from the multivariable Cox regression model, which included covariates from the univariable analyses with *P* < 0.10 to test the association between covariates. The proportional hazards assumption was checked. For the longitudinal data analysis, *P* values were calculated using repeated measures of two-way analysis of variance (ANOVA) on log-transformed data (time × group interaction term), and the sphericity assumption was checked using the Mauchly's test of sphericity. We then performed one-way ANOVA to test whether or not the difference between three time points were significant and post hoc Bonferroni tests to determine in which part of the longitudinal time period the “difference” occurred. Statistical analyses were performed with R software (R software, version 4.2.1).

## RESULTS

### Characteristics of the patients

We included 107 sepsis patients of whom the clinical characteristics are listed in Table 1. Among the total cohort, 82 (76.6%) patients had survived and 25 (23.4%) had died by day 28. For the subgroup of 63 (58.9%) patients with serial DDPCR results, the clinical characteristics are listed in Table S1.

**TABLE 1:** Clinical characteristics of the day-28 survivors and day-28 nonsurvivors of 107 patients^*e*^

	Survivors	Nonsurvivors	*P*
	(*N* = 82)	(*N* = 25)	
Gender, male	49 (59.8%)	19 (76.0%)	0.161
Age, categorical			0.224
<60	21 (25.6%)	4 (16.0%)	
60 to 70	25 (30.5%)	5 (20.0%)	
70 to 80	20 (24.4%)	6 (24.0%)	
80 to 100	16 (19.5%)	10 (40.0%)	
BMI	23.11 [21.61, 24.73]	23.37 [20.04, 24.86]	0.928
SOFA^*a*^ score	8 [5, 11]	9 [8, 13]	0.009
CCI^*b*^	4 [3, 6]	5 [4, 8]	0.022
Immunosupression	7 (8.5%)	3 (12.0%)	0.696
Catheter usage in past 48 h	33 (40.2%)	14 (56.0%)	0.176
Infection sites^*c*^			0.209
Singlar	61 (74.4%)	15 (60.0%)	
Multiple	21 (25.6%)	10 (40.0%)	
Count of microorganisms detected by DDPCR			0.000
1	61 (74.4%)	8 (32.0%)	
2	12 (14.6%)	13 (52.0%)	
3	4 (4.9%)	3 (12.0%)	
4	5 (6.1%)	0 (0%)	
5	0 (0%)	1 (4.0%)	
Count of microorganisms, binary			0.000
“mono” group	61 (74.4%)	8 (32.0%)	
“poly” group	21 (25.6%)	17 (68.0%)	
DNA load^*d*^, copies/mL	492 [128, 3011]	3,336 [126, 13,190]	0.146
White blood cells, ×10^9^ /L	12.2 [5.75, 17.31]	14.3 [6.9, 19.1]	0.675
Neutrophils, ×10^9^ /L	10.62 [4.78, 14.8]	13.5 [6.54, 17.3]	0.481
Lymphocytes, ×10^9^ /L	6.03 [4.14, 11.88]	5.01 [3.19, 6.86]	0.099
Monocytes, ×10^9^ /L	5.74 [2.64, 9.57]	3.37 [1.21, 6.62]	0.054
Platelets, ×10^12^ /L	130 [64, 228.5]	102 [33, 211]	0.265
C-reactive protein, mg/L	141.66 [82, 209]	139.76 [114.84, 188.2]	0.774
Procalcitonin, ng/mL	5.7 [1.13, 41.48]	1.88 [0.72, 12.75]	0.448
Total bilirubin, µmol/L	18.3 [12.8, 41.3]	11 [9.6, 20.6]	0.029
Serum creatinine, µmol/L	105 [65, 193]	170 [109, 262]	0.016
Lactate, mmol/L	1.95 [1.2, 2.9]	2.6 [1.65, 5]	0.027

^*a*^ SOFA = Sequential Organ Failure Assessment Score.

^*b*^ CCI = Charlson Comorbidity Index.

^*c*^ Infection sites were based on physicians' judgment at enrollment.

^*d*^ The DNA load of microorganisms detected by the DDPCR assay.

^*e*^ Data are presented as frequencies and percentages (%) for categorical data and medians and interquartile ranges [IQR] for continuous variables.

### Performance of the DDPCR assay

Of the 107 patients with positive DDPCR reports at enrollment, 106 had simultaneous BCs. A total of 90.9% (30/33) of the DDPCR tests identified pathogens detected by the 33 positive simultaneous BCs. However, DDPCR failed to detect the pathogens in three samples, one with an extra-panel *Aeromonas hydrophila* and two episodes with different pathogens reported by the simultaneous BCs, including *Staphylococcus hominis* (*n* = 1) and *Candida* spp. (*n* = 1). The remaining 73 samples were all tested negative by the synchronized BCs.

In the subgroup of 63 patients with three serial DDPCR tests, we found positive DDPCR results in 75.1% (142/189) tests, and 206 pathogens were identified in total (Table S3). Among them, *Klebsiella* spp. (*n* = 50), *Escherichia coli* (*n* = 49), and *Enterococcus* spp. (*n* = 25) were most commonly detected in the blood (Fig. S3), which was constant with the CHINET (15) report.

### Relationship between the initial DDPCR result and 28-day mortality

In the total cohort, 38/107 (35.5%) first DDPCR test reported more than one pathogen. According to the results of univariable Cox analyses, detection of poly-microorganisms by the first DDPCR, higher baseline Charlson Comorbidity Index (CCI) score, and higher baseline SOFA score were risk factors for 28-day mortality (Table 2). In further analysis, all covariates from the univariable analyses with *P* < 0.10 were included in the multivariate Cox proportional hazards regression model. Baseline CCI (adjusted HR = 1.14; 95% CI = 1.01–1.29; *P* = 0.041), SOFA score (adjusted HR = 1.18; 95% CI = 1.05–1.32; *P* = 0.005), and detection of poly-microorganisms (adjusted HR = 3.19; 95% CI = 1.34–7.62; *P* = 0.009) remained independent risk factors and the initial pathogen load was not a prognostic factor (adjusted HR = 1.17; 95% CI = 0.82–1.66; *P* = 0.385; Table 2).

**TABLE 2:** Results of the univariable and multivariable Cox regression analyses of 107 patients

Characteristic	Univariable analysis			Multivariable analysis		
	HR^*a*^	*95% CI^a^*	*P* value	Adjusted HR^*a*^	*95% CI^a^*	Adjusted *P* value
Age						
<60 years	—	—		—	—	
60–70 years	1.06	0.28, 3.94	0.933	1.01	0.27, 3.78	0.989
70–80 years	1.57	0.44, 5.55	0.487	1.40	0.39, 5.02	0.601
80–100 years	2.66	0.84, 8.50	**0.098^*d*^**	1.91	0.57, 6.43	0.298
BMI	1.04	0.94, 1.15	0.416	\	\	\
Gender				\	\	\
Men	—	—				
Women	0.51	0.20, 1.27	0.149			
CCI^*b*^	1.15	0.03, 1.28	**0.014^*d*^**	1.14	1.01, 1.29	**0.041^*d*^**
SOFA^*c*^	1.19	1.07, 1.31	**0.001^*d*^**	1.18	1.05, 1.32	**0.005^*d*^**
Infection sites				\	\	\
Single	—	—				
Multiple	1.73	0.78, 3.85	0.179			
Log10-transformed DNA load	1.35	0.99, 1.83	**0.056^*d*^**	1.17	0.82, 1.66	0.385
Count of microorganisms				\	\	\
= 1 (mono group)	—	—		—	—	
≥2 (poly group)	4.63	2.00, 10.76	**0.000^*d*^**	3.19	1.34, 7.62	**0.009^*d*^**

^*a*^ HR = Hazard Ratio; CI = Confidence Interval.

^*b*^ CCI = Charlson Comorbidity Index.

^*c*^ SOFA = Sequential Organ Failure Assessment Score.

^*d*^ The adjusted hazard ratio and 95% confidence intervals were from the multivariable Cox regression model with covariates from the univariable analyses with *P* < 0.10 (bold front) included.

### Relationship between dynamic pathogen load change and 28-day mortality

The 28-day mortality in the subgroup of 63 patients was 17.5% (11/63). By comparing the results of follow-up DDPCR at days 6–8 with the first one, patients can be divided into those with only decreased pathogen load (the “less” group; *n* = 40) and those with load increase or no decrease (the “not-less” group; *n* = 23). In the less group, 3/40 (7.5%) patients died before 28 days, while in the not-less group, a markedly high proportion of patients (8/23; 34.8%) died before 28 days (Fig. 2a). Kaplan–Meier univariable analysis also showed that, for the 63 people who were continuously monitored by DDPCR, no pathogen load decrease or load increase was a risk factor (*P* = 0.0082) for death before 28 days (Fig. 2b). The “poly” group also had higher all-cause mortality rate in this subgroup (Fig. 2c).

**Fig 3:**
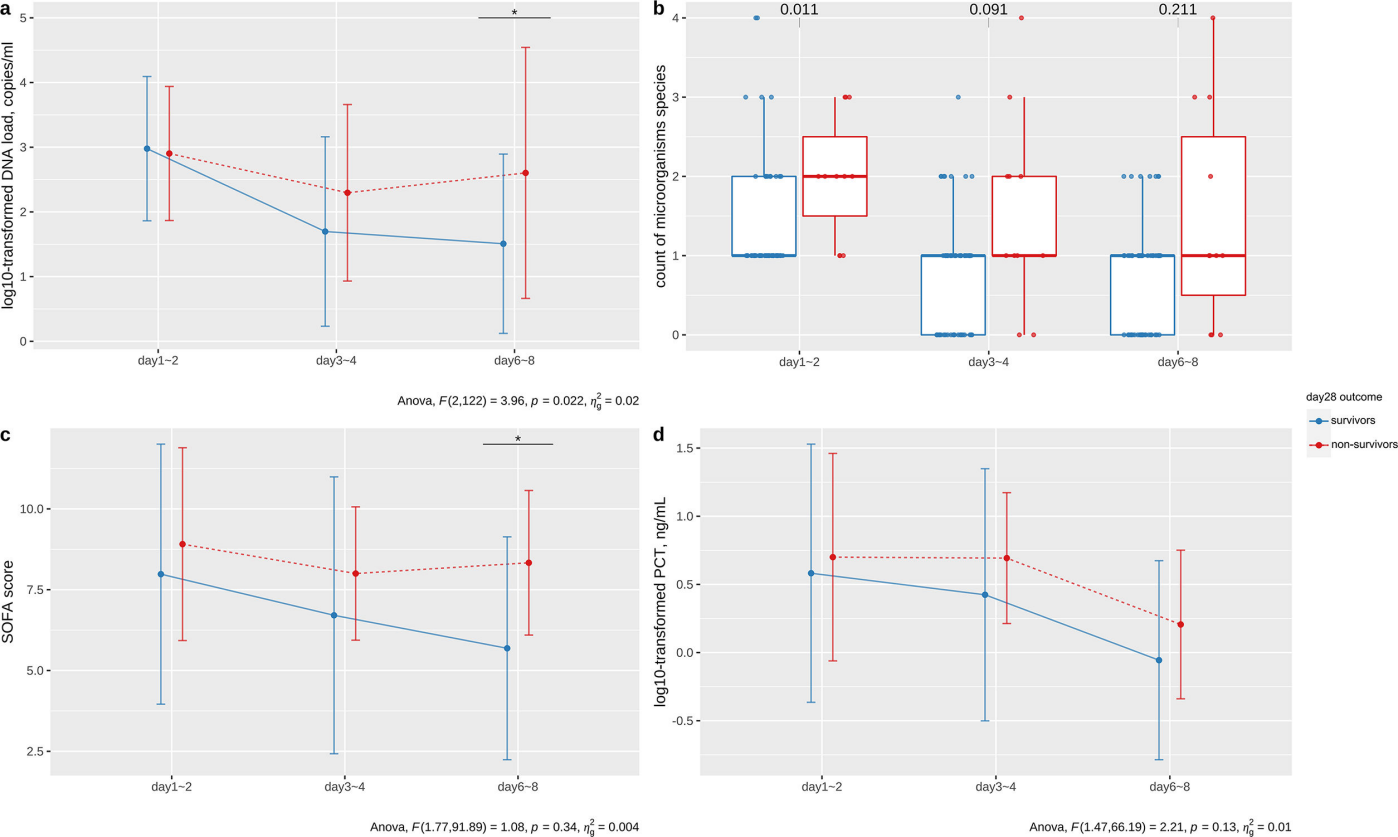
DNA load and count of microorganisms dynamically monitored with SOFA and PCT between day-28 survivors and nonsurvivors. (**a**) Log10-transformed DNA load by DDPCR, (**b**) count of microorganisms, (**c**) SOFA score, and (**d**) log10-transformed procalcitonin between survivors and nonsurvivors until day 28. Data are presented as mean and standard deviation error bar. For (a), (c), and (d), the *P* values in bottom-right corner were calculated using repeated measures two-way ANOVA on log-transformed data (time*group interaction term), and the asterisks on the picture represent *P* values of post-hoc Bonferroni tests. For (b), the Fisher's test *P* value was calculated and plotted at the top of picture.

The pathogen load changed in significantly different ways in 28-day survivors (*n* = 52) compared to nonsurvivors (*n* = 11; *P* = 0.022). There was an overall decline of pathogen load in patients who survived, whereas the load increased a week after sepsis diagnosis in nonsurvivors (Fig. 3a). Similar to the load kinetics, the SOFA score of the survivors tended to decrease continuously while there was no significant change in the nonsurvivors (Fig. 3c). Count of microorganisms and PCT continued to decline in both groups, but there was no significant difference in trends between survivors and nonsurvivors (Fig. 3b and d).

## DISCUSSION

Many studies have explored risk factors for poor prognosis in sepsis, including various clinical indicators such as serum lactate level and capillary refill time, treatment measures such as delayed application of antibiotics, as well as many developed severity scoring systems such as MEWS and SOFA score. However, it was still difficult to detect and monitor the changes of causative pathogen loads in a real-time way. Therefore, in this study, we applied a new DDPCR assay and aimed to explore its results' correlation with patient prognosis and the assay's application value when monitoring disease progression.

In our study, we found that poly-microorganisms were an independent risk factor for 28-day mortality after adjusting potential confounders including baseline SOFA score, CCI scores, and so on. In the clinical settings, poly-microorganisms detected by the DDPCR assay were found in 38/107 (35.5%) patients, which was similar to the pooled percentage (ranged from 0% to >60%) reported in a meta-analysis and several other studies (13, 16). The conclusion was in line with some previous studies (17, 18). Pavlaki et al. (18) reported that the all-cause 28-day mortality was higher in patients with polymicrobial bloodstream infections (pBSIs) versus monomicrobial BSI (38.3% vs 24.7%; *P* = 0.033) despite comparable appropriateness of treatment (78.7% vs 86.6%), and pBSI (HR = 1.86; *P* = 0.039) was a significant contributor to 28-day mortality in multivariate analysis. However, some studies reported no significant difference (13). One meta-analysis found that polymicrobial infection was associated with lower 28-/30-day mortality [odds ratio (OR) = 0.78; 95% CI = 0.54–1.12; I2 = 48%), though the authors suggested that lack of a clear definition for polymicrobial infection was a common limitation in many studies.

Studies showed different results on whether the initial pathogen load was correlated with mortality. Kirkbright et al. (19) stated that the bacterial 16s rDNA correlated with the mortality in patients suffering from *E. coli* and *Staphylococcus aureus* bacteremia, and Ho et al. (20) found that a high initial serum *S. aureus* load was associated with sepsis mortality. Dickson et al. (21) used DDPCR to quantitatively detect the bacterial 16s rRNA load in the lungs of ICU patients on admission and indicated that patients with increased bacterial load burden and enrichment of the lung microbiome suffered poorer ICU outcomes. Pérez-Nadales et al. (22) have reported the predictive value of the bacterial load of KPC-producing *Klebsiella pneumoniae* within the intestinal microbiota in mortality. In viral diseases, Li et al. (23) and Chen et al. (24) reported that the viral load in patients with SARS-CoV-2 viremia predicted the outcomes. Also, for lymphomas and oropharyngeal cancer, whole blood Epstein–Barr virus load could predict prognosis (25), so did HPV16 load in anal cancer (26). However, some studies showed opposite results that Ziegler et al. (27) and Shao et al. (13) did not find significant association between the initial pathogen load and sepsis mortality. Besides, we did notice that most of the previous studies were targeting a certain single molecule. When it comes to the quantitative results of multiplex PCR, more exploration on how to describe the load feature are needed.

With the advancement of quantitative techniques, simultaneous detection and monitoring of multiple pathogens is becoming a reality. We strengthened the importance of pathogen load surveillance and have explored that increased serum pathogen loads were associated with poor prognosis. The results could be supported by a few other studies focusing on dynamic changes monitored by quantitative assays, mainly real-time PCR assays. In a prospective observational study, which used the rate of bacterial clearance determined by real-time PCR to evaluate the appropriateness of antibiotic prescription in 51 critical patients with *Acinetobacter baumannii* bacteremia, slower rate of bacterial clearance was found to be a risk factor for in-hospital mortality (OR = 2.32; *P* = 0.04) (28). The prospective study by Ho et al. (20) reported that mean *mecA* DNA levels detected by a quantitative real-time PCR assay in survivors tended to decline consistently and high levels of *mecA* load were associated with mortality.

Our study is the largest cohort of sepsis patients in which the prognostic value of pathogen species and load has been evaluated. One of the limitations is that we divided the infection types into pBSIs and monomicrobial BSIs, rather than looking into the specific species composition. Also, we have not taken the specific infection sites as primary causes of BSIs into consideration.

Our prospective cohort uncovered potential value for pathogen quantification in monitoring the sepsis progression by demonstrating that detection of poly-microorganisms and increased serum pathogen load indicated the 28-day poor prognosis. Further studies focusing on the mechanisms of host immunogenicity effect on the pathogen load dynamics (29) may be needed in sepsis patients.

## SUPPLEMENTAL MATERIAL

Supplemental legend spectrum.02558-23-s0001.docx Legend for Figure S1.

Table S1 spectrum.02558-23-s0002.docx Clinical characteristics of the day-28 survivors and non-survivors of 63 patients.

Table S2 spectrum.02558-23-s0003.docx The pathogen species and antimicrobial genes targeted by the DDPCR assay.

Table S3 spectrum.02558-23-s0004.docx The pathogens detected by the DDPCR assay.

Figure S1 spectrum.02558-23-s0005.pdf Frequency of pathogens detected by the DDPCR assay.

ASM does not own the copyrights to Supplemental Material that may be linked to, or accessed through, an article. The authors have granted ASM a non-exclusive, world-wide license to publish the Supplemental Material files. Please contact the corresponding author directly for reuse.
