# Supplementary material for: Prognostic value of poly-microorganisms detected by droplet digital PCR and pathogen load kinetics in sepsis patients: a multi-center prospective cohort study
Source: Microbiol Spectr. 2024 Mar 25;12(5):e02558-23. doi: 10.1128/spectrum.02558-23 (PMC11064489; doi:10.1128/spectrum.02558-23)
Supplement: Supplemental legend — Legend for Figure S1. [file spectrum.02558-23-s0001.docx]

**Figure S1. Frequency of pathogens detected by the DDPCR assay.**Pathogen distribution of 3 serial DPCR results is represented using 3 bar charts, and the number at the top of each chart indicates the time of the test (day0, 3 and 7). We found positive DDPCR results in 75.1% (142/189) tests and 206 pathogens were identified in total. The 189 DDPCR tests were from the subgroup of 63 patients with 3 serial DDPCR tests(figure 1).
