# Supplementary material for: Prognostic value of poly-microorganisms detected by droplet digital PCR and pathogen load kinetics in sepsis patients: a multi-center prospective cohort study
Source: Microbiol Spectr. 2024 Mar 25;12(5):e02558-23. doi: 10.1128/spectrum.02558-23 (PMC11064489; doi:10.1128/spectrum.02558-23)
Supplement: Table S1 — Clinical characteristics of the day-28 survivors and non-survivors of 63 patients. [file spectrum.02558-23-s0002.docx]

Table S1. Clinical characteristics of the day28 survivors and non-survivors of 63 patients

| **characteristics** | **0** | **1** | **P-value** |
| --- | --- | --- | --- |
|  | **(N=52)** | **(N=11)** |  |
| Gender, male | 37 (71.2%) | 8 (72.7%) | 1 |
| Age,categorical |  |  | 0.1714 |
| <60 | 18 (34.6%) | 1 (9.1%) |  |
| 60to70 | 14 (26.9%) | 3 (27.3%) |  |
| 70to80 | 11 (21.2%) | 2 (18.2%) |  |
| 80to100 | 9 (17.3%) | 5 (45.5%) |  |
| BMI | 23.14 [21.48, 25.06] | 22.49 [20.7, 25.09] | 0.8152 |
| SOFA*^1^* score | 7 [5, 11] | 9 [7.5, 10] | 0.3535 |
| CCI*^2^* | 4 [2.75, 6] | 6 [5, 10] | 0.0067 |
| Immunosupression | 4 (7.7%) | 1 (9.1%) | 1 |
| Catheter usage in past 48h | 19 (36.5%) | 7 (63.6%) | 0.1757 |
| Invasive operation in past 48h | 8 (15.4%) | 3 (27.3%) | 0.3888 |
| Infection sites*^3^* | 15 (28.8%) | 7 (63.6%) | 0.0394 |
| singlar | 37 (71.2%) | 4 (36.4%) |  |
| multiple | 15 (28.8%) | 7 (63.6%) |  |
| Count of microorganisms by DDPCR |  |  | 0.0085 |
| 1 | 38 (73.1%) | 3 (27.3%) |  |
| 2 | 9 (17.3%) | 5 (45.5%) |  |
| 3 | 3 (5.8%) | 3 (27.3%) |  |
| 4 | 2 (3.8%) | 0 (0%) |  |
| Count of microorganisms, binary |  |  | 0.0114 |
| mono | 38 (73.1%) | 3 (27.3%) |  |
| poly | 14 (26.9%) | 8 (72.7%) |  |
| DNA load by DDPCR*^4^* | 438.1 [144, 5400] | 445 [115, 3391] | 0.9279 |
| White blood cells, ×10^9/L | 11.3 [5.55, 15.95] | 13.98 [9.8, 18.39] | 0.4555 |
| Neutrophils, ×10^9/L | 8.61 [4.6, 13.38] | 11.64 [8.19, 16.15] | 0.5647 |
| Lymphocytes, ×10^9/L | 5.92 [3.52, 11.8] | 4.68 [2.92, 9.51] | 0.3247 |
| Monocytes, ×10^9/L | 6.47 [2.73, 9.44] | 4.81 [2.05, 8.7] | 0.5191 |
| Platelets, ×10^12/L | 6.47 [2.7, 9.4] | 4.81 [2.1, 8.7] | 1 |
| C-reactive protein, mg/L | 141.66 [74, 210.5] | 136 [108.37, 145.02] | 0.3837 |
| Procalcitonin, ng/mL | 4.59 [0.6, 30] | 9.29 [0.9, 12.75] | 0.7072 |
| Total bilirubin, µmol/L | 19.8 [15.72, 46.47] | 10.4 [9.15, 17.1] | 0.0348 |
| Serum creatinine, µmol/L | 92 [63.5, 157.5] | 161 [88, 181.5] | 0.311 |

*^1^SOFA= Sequential Organ Failure Assessment Score; ^2^ CCI= Charlson Comorbidity Index; ^3^infection sites were based on physicians’ judgement at enrollment; ^4^the DNA load of microorganisms detected by the DDPCR assay. Data are presented as frequencies and percentages (%) for categorical data, and medians and interquartile ranges [IQR] for continuous variables.*
