# Supplementary material for: Prognostic value of poly-microorganisms detected by droplet digital PCR and pathogen load kinetics in sepsis patients: a multi-center prospective cohort study
Source: Microbiol Spectr. 2024 Mar 25;12(5):e02558-23. doi: 10.1128/spectrum.02558-23 (PMC11064489; doi:10.1128/spectrum.02558-23)
Supplement: Table S2 — The pathogen species and antimicrobial genes targeted by the DDPCR assay. [file spectrum.02558-23-s0003.docx]

Table S2. The pathogen species and antimicrobial genes targeted by the DDPCR assay

| **Panel** | **species** |
| --- | --- |
| 1 | *Pseudomonas aeruginosa* |
|  | *Escherichia coli* |
|  | *Klebsiella* spp. |
|  | *Acinetobacter baumannii* complex |
|  | *Enterobacter cloacae* |
| 2 | *Staphylococcus aureus* |
|  | *Enterococcus* spp. |
|  | Coagulase-negative staphylococci |
|  | *Streptococcus* spp. |
|  | *Candida* spp. |
| 3 | *Stenotrophomonas maltophilia* |
|  | *Citrobacter* spp. |
|  | *Serratia marcescens* |
|  | *Proteus mirabilis* |
|  | *Burkholderia cepacia* complex |
| 4 | blaKPC |
|  | mecA |
|  | OXA-48 |
|  | NDM、IMP |
|  | VanA、VanM |
