# Supplementary material for: Prognostic value of poly-microorganisms detected by droplet digital PCR and pathogen load kinetics in sepsis patients: a multi-center prospective cohort study
Source: Microbiol Spectr. 2024 Mar 25;12(5):e02558-23. doi: 10.1128/spectrum.02558-23 (PMC11064489; doi:10.1128/spectrum.02558-23)
Supplement: Table S3 — The pathogens detected by the DDPCR assay. [file spectrum.02558-23-s0004.docx]

Table S3. The pathogens detected by the DDPCR assay

| **species** | **count of appearance by DDPCR** |
| --- | --- |
| *Pseudomonas aeruginosa* | *8* |
| *Escherichia coli* | *49* |
| *Klebsiella* spp. | *50* |
| *Acinetobacter baumannii* complex | *16* |
| *Enterobacter cloacae* | *1* |
| *Staphylococcus aureus* | *2* |
| *Enterococcus* spp. | *25* |
| Coagulase-negative staphylococci | 14 |
| *Streptococcus* spp. | *24* |
| *Candida* spp. | *14* |
| *Stenotrophomonas maltophilia* | *3* |
| *Citrobacter* spp. | *0* |
| *Serratia marcescens* | *0* |
| *Proteus mirabilis* | *0* |
| *Burkholderia cepacia* complex | *0* |
