# Supplementary figures and images for: Prognostic value of poly-microorganisms detected by droplet digital PCR and pathogen load kinetics in sepsis patients: a multi-center prospective cohort study
Source: Microbiol Spectr. 2024 Mar 25;12(5):e02558-23. doi: 10.1128/spectrum.02558-23 (PMC11064489; doi:10.1128/spectrum.02558-23)

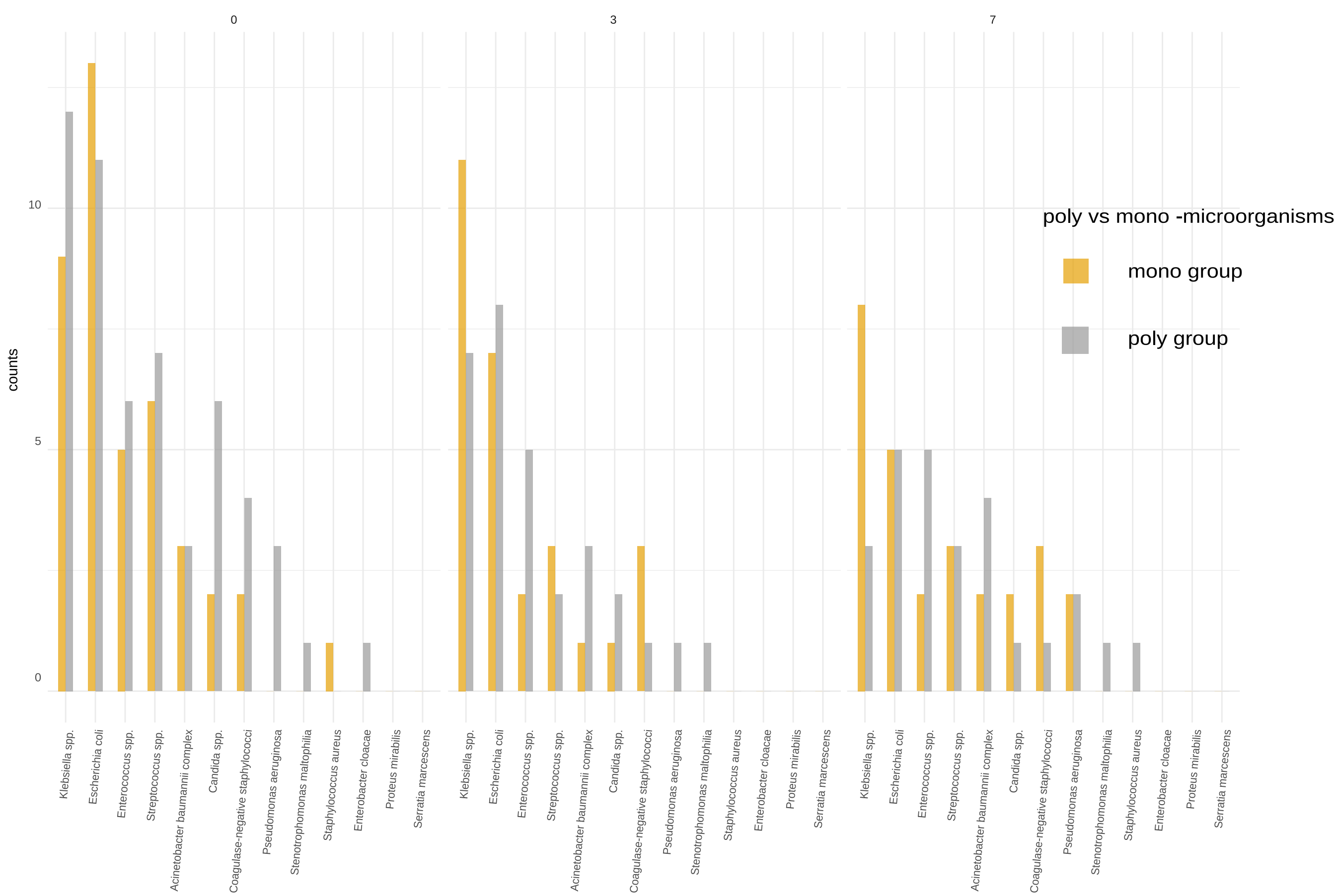

Supplement: Figure S1 — Frequency of pathogens detected by the DDPCR assay. [file spectrum.02558-23-s0005.pdf]
